# CT-based comparison of porcine, ovine, and human pulmonary arterial morphometry

**DOI:** 10.1038/s41598-023-47532-8

**Published:** 2023-11-18

**Authors:** Leonid Goubergrits, Marie Schafstedde, Nikola Cesarovic, Angelika Szengel, Boris Schmitt, Moritz Wiegand, Jan Romberg, Andreas Arndt, Titus Kuehne, Jan Brüning

**Affiliations:** 1 Deutsches Herzzentrum Der Charité (DHZC), Institute of Computer-Assisted Cardiovascular Medicine, Berlin, Germany; 2 Charité – Universitätsmedizin Berlin, corporate member of Freie Universität Berlin and Humboldt-Universität Zu Berlin, Berlin, Germany; 3 Department of Health Sciences and Technology, ETH Zurich, Zurich, Switzerland; 4 Biotronik, Berlin, Germany; 5 1000Shapes GmbH, Berlin, Germany

**Keywords:** Computational biology and bioinformatics, Anatomy, Cardiology

## Abstract

To facilitate pre-clinical animal and in-silico clinical trials for implantable pulmonary artery pressure sensors, understanding the respective species pulmonary arteries (PA) anatomy is important. Thus, morphological parameters describing PA of pigs and sheep, which are common animal models, were compared with humans. Retrospective computed tomography data of 41 domestic pigs (82.6 ± 18.8 kg), 14 sheep (49.1 ± 6.9 kg), and 49 patients (76.8 ± 18.2 kg) were used for reconstruction of the subject-specific PA anatomy. 3D surface geometries including main, left, and right PA as well as LPA and RPA side branches were manually reconstructed. Then, specific geometric parameters (length, diameters, taper, bifurcation angle, curvature, and cross-section enlargement) affecting device implantation and post-interventional device effect and efficacy were automatically calculated. For both animal models, significant differences to the human anatomy for most geometric parameters were found, even though the respective parameters’ distributions also featured relevant overlap. Out of the two animal models, sheep seem to be better suitable for a preclinical study when considering only PA morphology. Reconstructed geometries are provided as open data for future studies. These findings support planning of preclinical studies and will help to evaluate the results of animal trials.

## Introduction

Animal models play a crucial role in biomedical research including basic research focussing on identifying aetiology and mechanisms of diseases, or development of medical devices. According to regulatory requirements they currently still are considered a necessary pre-clinical tool on the road to the potential translation towards clinical trials^1,2^. However, proper selection of the animal model, considering different aspects, such as the similarity of the anatomical region of interest with respect to shape and size, which for example is important for the appropriate modelling of device implantation procedures, is necessary.

The success of the implantation procedure and the subsequent proper function of cardiovascular implantable devices is affected by both the anatomical structure as well as the cardiovascular function and will also affect both anatomy and function in turn. This complex interaction between implanted medical devices and the cardiovascular system can be associated with risks in both directions. The device might affect the proper cardiovascular function, as for example coronary compression and valve embolization reported after transcatheter pulmonary valve replacement^3^, pulmonary artery (PA) rupture reported after transcatheter pulmonary valve implantation^4^, or device-related complications reported for pulmonary artery pressure sensors (PAPS)^5^.

In frames of the H2020 project SIMCor (https://www.simcor-h2020.eu), a platform for in-silico assessment of device effect and efficacy of novel or existing cardiovascular implantable devices is developed. Here, one use case are PAPS for monitoring of heart failure (HF) patients, such as the CardioMEMS HF System^6^ or the Cordella HF System^5^. PAPSs are designed to be implanted via a catheter into the left (LPA) or right pulmonary artery (RPA). Thus, they are class III medical devices according to both the Federal Drug Administration medical device classification as well as the European Medical Device Regulation, requiring the highest level of testing and safety evaluation. This evaluation usually includes animal experiments using large animal models such as pigs or sheep. Here, an insufficiently answered question is, to which extent findings from animal experiments can be translated towards human trials, and where animal experiments fail to provide sufficient similarity to human anatomy and function.

Studies on human PA morphometry, including intra-acinar and pre-acinar arteries, date back to the 1970s as noted in Dong et al.^7^. PA morphometry as well as PA hemodynamics are crucial for understanding of the pulmonary circulation with and without vascular diseases such as pulmonary arterial hypertension. However, the focus of these studies was to better understand tree-like structure of the PA, which is usually sorted into diameter-defined Strahler numbers aiming at quantification of branching complexity, by analysing relationships of vessel segment diameters, lengths, tortuosity, and bifurcations. Also, the relationship of these parameters with patient metrics such as body surface area (BSA), age, or height, as well as applicability of Murray´s law, which describes the volume flow distribution in vascular bifurcations, was investigated earlier^7^. These studies were also used to generate geometric models of the PA to investigate the role of the vessel branching structure on flow distribution^8^. Similar morphometric studies were also performed for different animal species including analysis of the ovine^9^ or porcine PA^10,11^. Information on PA morphometry collected in these studies is helpful for the initial selection of animal models but not entirely sufficient for description of the morphometric variation relevant for in-silico analysis of implantable cardiovascular devices. The studies comparing different species’ anatomies focus either on perfusion^9^ aspects or the similarity with respect to the branching structure of the different vessel generations^10^, but not the anatomic details of the vessels posing as landing zone for PAPS and are thus not entirely sufficient for the PAPS development. While other studies describe the porcine anatomy as well as the weight-dependent changes in anatomical sizes in detail^11^, relevant aspects of the complex 3D anatomy and the comparison against human anatomy are not available yet.

However, both for improving the translation of findings made in animal experiments as well as for the proper design of in-silico models for device evaluation, better understanding of the similarities between the PA anatomy of different species is necessary. The anatomy of the PA affects both the implantation as well as the function of a PAPS. Most importantly, the animal model must be sufficiently similar with respect to the target implantation site and its geometric properties, such as lengths, diameters, and configurations of branching vessels. This is necessary, as aspects of the device, such as size of the sensor, as well as size, shape and strength of the fixation elements are designed with respect to the human use.

If relevant differences in the implantation site anatomy are present, the device performance in the animal model might be different than compared to the clinical use. For example, larger diameters of the implantation site will result in weaker fixation, increasing the risk of device displacement, whereas smaller diameters will result in larger contact stresses, increasing the risk for vessel perforation. However, also more complex shape parameters are of interest both for the implantation but also for proper device function. For example, implantation in a strongly curved vessel might prevent the sensor to be adjacent to the vessel wall, which in turn might result in unfavourable hemodynamic conditions, such as stagnation or flow separation zones. If the vessels feature stronger taper, meaning that they decrease in diameter fast, the distal part of the sensor might block a relevant portion of the vessel cross-section, causing flow acceleration and increased shear rates. Finally, some morphometric aspects of the pulmonary artery might affect the intra-arterial function even without the sensor implantation. For example, the cross-sectional enlargement of the main PA (MPA) bifurcation and the angle of this bifurcation, might result in relevant differences in hemodynamics, and therefore might encumber translation of thrombogenesis experiment in large animal models. A set of investigated geometric parameters including a motivation behind this selection is summarized in the Table 1 (see Materials and Methods).

**Table 1:** Summary of geometric parameters selected for this study and respective motivations.

Parameter	Motivation
Number of side branches	(1) Side branches can affect the implantation procedure since fixation wires could deploy into the side branches, resulting in skewed device implantation positions (2) Side branches near the implanted sensor covering of side branches by the sensor can results in complex, disturbed flows causing thrombus formation
Diameter	(1) Larger diameters of the landing site are associated with less secure fixation, whereas lower diameters can result in higher degrees of the stenosis due to the vessel cross-section being reduced by the device (2) At a fixed volumed flow rate, larger vessel diameters are associated with lower WSS, which are known to promote thrombus formation, whereas the sensor is more likely to cause flow separation and recirculation regions in smaller vessels, also promoting thrombus formation
Length	Larger vessel lengths will result in higher probabilities to identify a suitable the landing zone for the device without side branches
Bifurcation angle	Larger bifurcation angles are associated with flow disturbance (flow separation and recirculation zones) due to the change of the flow direction, which again can promote thrombus formation
Vessel taper	The vessel taper T was defined as T = (D_in_ − D_out_)/L, where L is the vessel segment length and D_in_ with D_out_ are vessel segment diameters at the vessel segment inlet and outlet. The vessel tapering aims to compensate WSS reduction due to flow rate reduction caused by side branches. Higher tapering accelerates flow thus reducing the danger of flow separation
Curvature index CI	The curvature index (CI) of the LPA or RPA vessel is defined as the ratio of the length of the line connecting start- and endpoints of the vessel segment centreline and the length of the real, curved centreline. A curvature index of the 1 means that the vessel is characterised by a straight centreline. The lower the curvature index, the higher the vessel curvature, which can cause flow separation and recirculation regions. Furthermore, a strongly curved vessel means that it is more challenging to deploy the sensor in a position that is entirely parallel to the vessel wall
Enlargement index EnI	The enlargement index (EnI) was defined as EnI = (1 − ((D_LPA_^2^ + D_RPA_^2^)/D_MPA_^2^))^2^, where D is the diameter of the MPA, LPA and RPA, respectively. This function is proposed in technical fluid mechanics to describe the pressure loss coefficient in a sudden expansion due to formation of recirculation regions^21^. Like the curvature index, higher enlargement indices are associated with disturbed flow conditions

In this study, we focus on assessing similarities and differences with respect to these morphometric size and shape parameters between the different species.

## Materials and methods

To compare the pulmonary artery morphometry of the two animal models with that of humans, retrospective computed tomography (CT) data available from previous animal studies as well as clinical routine was used. PAPS can usually be implanted in both the left and right PA, even though manufacturer recommendation might favor a dedicated implantation site. Spatial dimensions of current PAPS systems are 15.0 mm × 3.4 mm × 2.0 mm (CardioMEMS) and 19.3 mm × 3.8 mm × 1.9 mm height (Cordella) in length, width and height respectively. Target diameters of the implantation site for the CardioMEMS device are between 7 and 11 mm^12^ and between 12 and 26 mm for the Cordella system^13^, respectively. For fixation of the device within the pulmonary artery wires are attached to the sensor body. An additional, a novel device with sizes similar to those of the CardioMEMS and Cordelia systems will also be evaluated, for which the target diameters of the implantation site are estimated to lie between 9 and 14 mm as optimal. This defined the domain of interest for the anatomical study, which included the MPA, LPA, and RPA as well as all side branches of the LPA and RPA. 3D geometries of the PA were reconstructed from available CT image data aiming at reconstruction of the vessels until LPA and RPA diameters were below 8 mm or as long as the image data allowed.

### Imaging data

Data of 41 domestic pigs (Swiss Large White, 3 castrated males, 38 females), weighing 82.6 ± 18.8 (range 52–117 kg) kg, and being 3–6 months old, that underwent pre-operative cardiac CT for a different project in the Department of Health Sciences and Technology, ETH Zürich, were re-used in this study. Acquisition was done by dual-source multi-slice spiral CT scanner SOMATOM Definition Flash (Siemens Healthineers, Forchheim, Germany) with 120 kV tube voltage as well as (0.391–0.684 mm) × (0.391–0.684 mm) in-plane resolution and (0.3 mm) slice thickness. These animal studies were approved by the local Committee for Experimental Animal Research (Cantonal Veterinary Office Zurich, Switzerland) under the approval numbers 152/2013, 219/2016 and 138/2017. More information about the experiment for which the CT information was acquired can be found in the respective studies^11,14^.

Additionally, pre-operative CT image data of 14 sheep (breeds: Ovis aries, German heath sheep; 7 males, 7 females) weighing 49.1 ± 6.9 (range 40 – 63 kg) kg, and being of 9.0 ± 0.8 months old, were available from a project aiming at development of a pulmonary valve prosthesis. Dual-source multi-slice spiral CT scanner SOMATOM Definition Flash (Siemens Healthcare GmbH, Erlangen, Germany) with 100 kV tube voltage, (0.666 – 0.977 mm) × (0.666–0.977 mm) in-plane resolution and (0.7 mm) slice thickness was used for data acquisitions. This study was approved by the State Office of Health and Social Affairs Berlin and conducted according to the Federation of Laboratory Animal Science Associations guidelines. More information about the CT imaging procedure can be found in the respective study^14^. Both animal experiments from which this data were derived were performed in accordance with all relevant guidelines and regulations. This study exclusively re-uses existing imaging data of animals that was acquired in earlier studies and were not related to this research at all.

Finally, retrospective CT image data of 49 aortic stenosis patients, weighing 76.8 ± 18.2 kg, with an age distribution of 81 ± 7.6 years (median 82.5 years and interquartile range (IQR) of [77.75–85.0]), and a female percentage of 60% were used for reconstruction of human pulmonary artery samples. CT data sets of the entire heart were acquired as part of transcatheter aortic valve implantation (TAVI) planning between February 2019 and October 2020 at our clinical centre. CT data were acquired with wide area-detector volume CT scanners: Aquilion One Vision (Canon Medical Systems, Tochigi, Japan) or Revolution CT (GE Healthcare, Chicago, IL, USA) with 100 kV tube voltage, (0.390–0.648 mm) × (0.390–0.648 mm) in-plane resolution and (0.5–1.0 mm) slice thickness. The use of retrospective data for this study was approved by the institutional review board (Ethikkommission Charité–Universitätsmedizin Berlin, approval number EA2/004/21). Individual informed consent was waived by the IRB due to the retrospective nature of this study. Research using this data was performed in accordance with all relevant guidelines and regulations. Additional information on the CT acquisition and the data set can be found in the respective study^15^.

Imaging procedures for both sheep and pigs were performed under general anaesthesia. Pigs were sedated using an intramuscular injection of ketamine (Ketasol®-100 ad us.vet.; Dr. E. Graeub AG, Berne, Switzerland; 15 mg/kg body weight), azaperone (Stresnil® ad us.vet.; Elanco Tiergesundheit AG, Basel, Switzerland; 2 mg/kg body weight) and atropine (Atropinsulfat KA vet 0.1%; Kantonsapotheke, Switzerland; 0.05 mg/kg body weight). Anesthesia was induced by an intravenous administration of propofol (Propofol ®- Lipuro 1%, B. Braun Medical AG; Sempach, Switzerland; 1–2 mg/kg body weight) to achieve relaxation and swallow-reflex diminishment sufficient for intubation. Anesthesia was maintained with propofol (5–10 mg/kg/h). Sheep were sedated using intravenous thiopental 7.5–10 mg/kg (Trapanal® 0.5 g, Inresa Arzneimittel GmbH, Germany) and propofol 1–2.5 mg/kg (Propofol-Lipuro 1%®, Braun, Melsungen, Germany). The sheep were intubated, and a gastric tube was placed into the rumen for gas evacuation. Total intravenous anesthesia was performed using propofol (2.5–8.0 mg/kg/h) and ketamine (2–5 mg/kg/h). All CT images were acquired before any further intervention, such as device implantation, and no further medication was administered.

### Geometry reconstruction

CT image data was used to reconstruct the end-diastolic 3D geometry of the PA for all three species. The reconstruction was performed using ZIBAmira (v. 2015.28, Zuse Institute Berlin, Germany). In this study, mostly manual segmentation procedures were used. To support the manual segmentation, first, all image voxels above a specific Hounsfield Unit (HU) threshold were labelled as potential PA lumen. The threshold definition was individual due to high variability in the contrast agent concentration between the different data sets. In ovine image data, the HU threshold varied between 170 and 400. In porcine image data, the HU threshold varied between 100 and 250, whereas in human data the HU threshold varied between 80 and 190.

**Figure 1:**
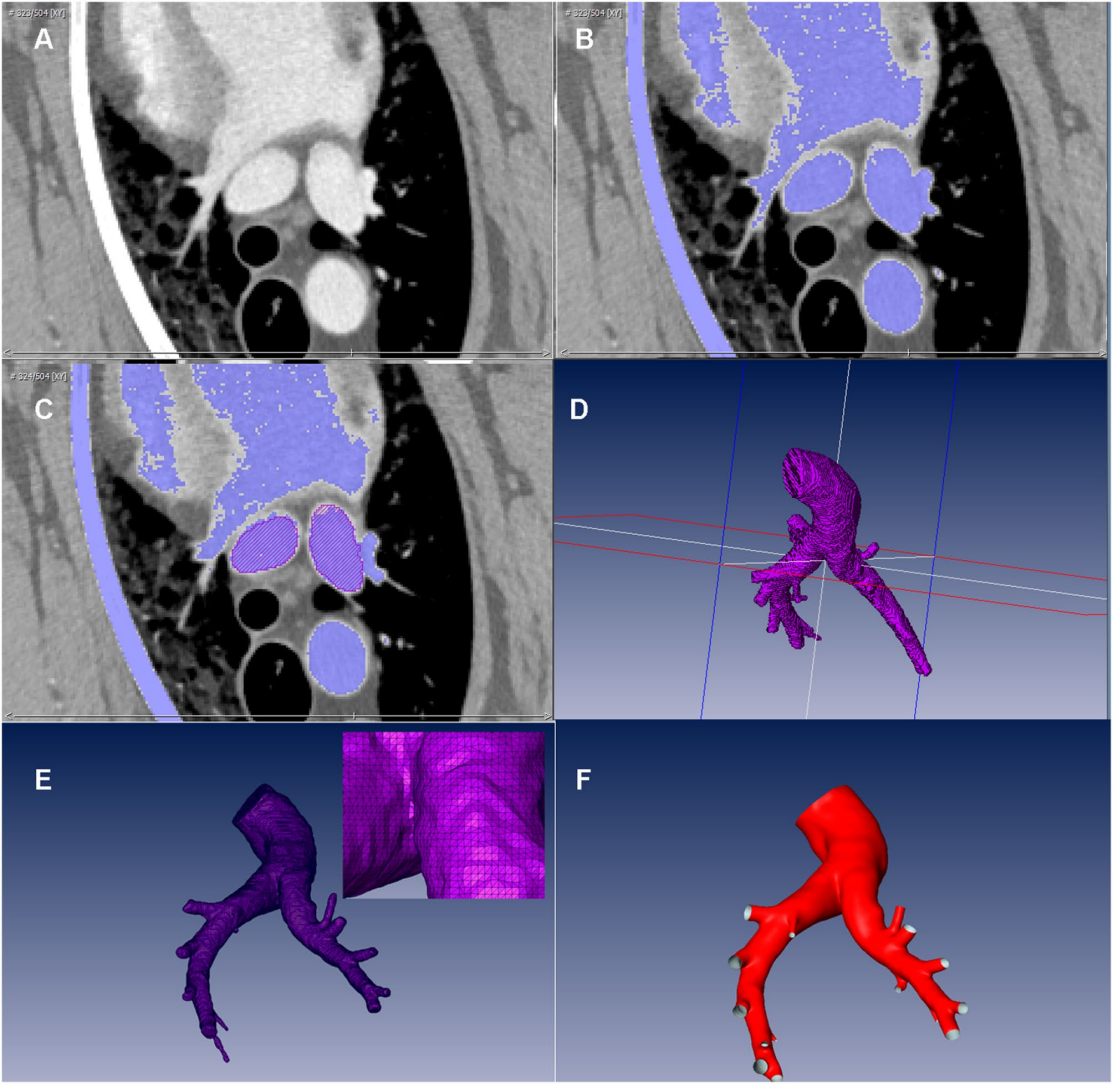
Geometry reconstruction workflow: (**A**) Exemplary orthogonal slice of the CT data of a sheep. (**B**) Blue colour highlights the automated masking of all voxels in the slice with Hounsfield Unit values above 175. (**C**) Red areas indicate the PA lumen in the slice after the manual correction. (**D**) 3D voxel field of the PA. (**E**) Rough triangulated surface reconstruction. (**F**) Final surface after smoothing.

**Figure 2:**
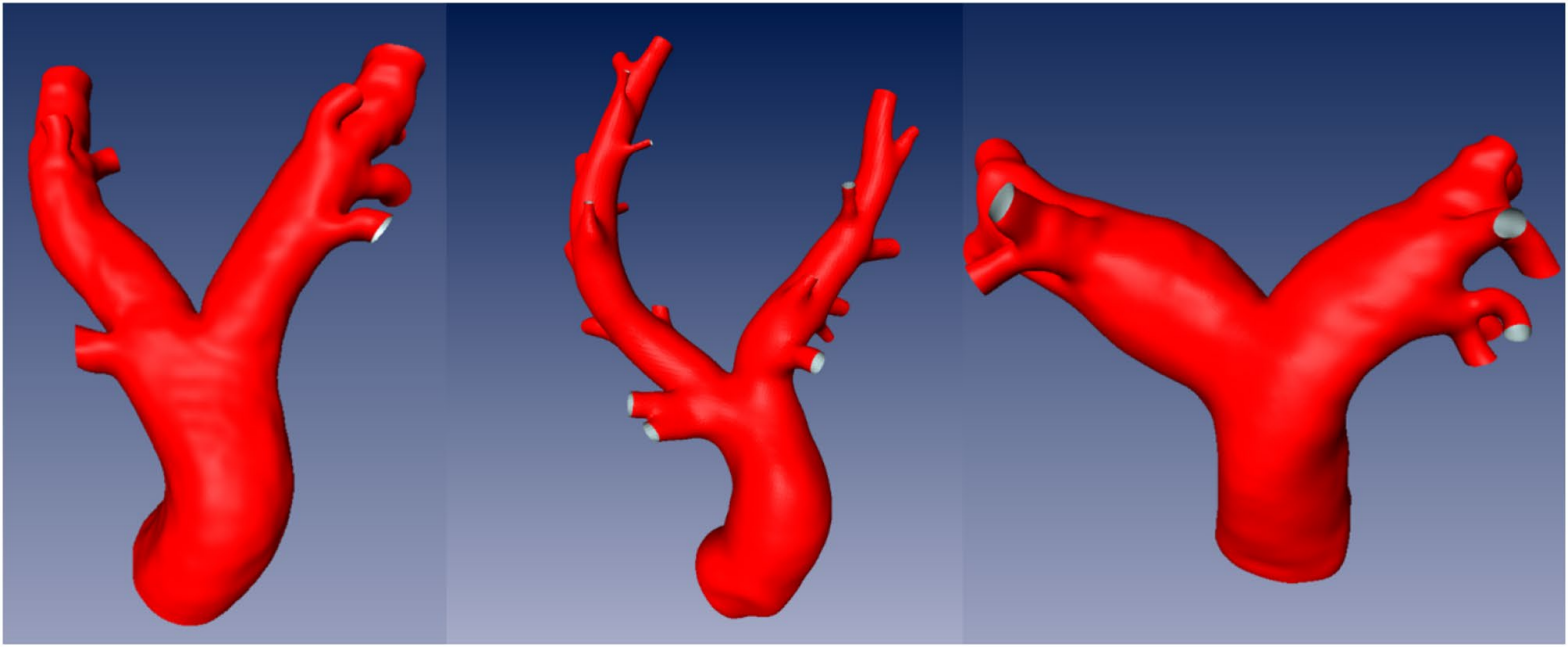
Exemplary surfaces of the porcine (left), ovine (middle), and human (right) pulmonary arteries.

From these image voxel candidates, the PA lumen was reconstructed slice by slice, beginning from right ventricular outflow tract (RVOT), using brush, flood fill, as well as the region-growing tools. The segmentation was corrected by slicing through the data stack from top to bottom as well as from left to right and front to back. The voxel label field was then used to generate rough triangulated surface using a Marching Cubes algorithm. Afterward, all geometries were smoothed using a volume-preserving smoothing algorithm^16^ implemented in JavaView. Smoothing of the surface geometries was necessary, as the discrete resolution of CT images results in surface mesh geometries with pronounced steps. This procedure is illustrated in Fig. 1. All geometries were truncated at the MPA directly after the sinus of the pulmonary trunk, whereas all side branches of the LPA and RPA were truncated at the approximately 10 mm length from their orifices. Exemplary reconstructions for all three species are shown in Fig. 2.

### Operator-bias analysis for the surface reconstruction

Since image reconstruction was performed manually, the results are possibly affected by inter-operator variability. To quantify the impact of this potential bias, a sub-cohort of 10 patients was randomly selected for an operator bias analysis (weight: 83 ± 21.1 kg weight, age: 81 ± 4.8 years, 70% female). These data sets were reconstructed by two experienced operators who both had more than 10 years of experience in image-based reconstruction of different cardio-vascular structures. Both operators reconstructed all 10 human PA, and the resulting anatomical measurements were statistically compared.

To quantify differences between different reconstructions of the same PA, two approaches were used. First, we assessed surface differences using, by calculating the Euclidean distance for each vertex of the triangulated surface mesh of one surface to the nearest point of the second surface. During the reconstruction procedure, no alignment, transformation, or scaling is performed. Therefore, no registration of the two independent surface reconstructions was necessary. The mean and standard deviation (SD) of these distances were then calculated for a confined region including only the MPA, LPA and RPA, to avoid impact of differences in segmentation of the length of branching vessels, which were of no importance for this investigation.

### Geometric parameters of interest

The in-silico platform for assessment of device effect and efficacy simulation for PAPS aims to model and predict three clinically relevant aspects: (1) interaction between blood flow and the device to assess the risk for thrombus formation; (2) device displacement, for which the patient-specific anatomy as well as the hemodynamic forces acting on the device are of interest; (3) risk for perforation of the vessel wall by the device fixation.

These aspects are all, to varying degree, affected by the PA anatomy and thus were relevant for the decision of which anatomical parameters to assess. Modelling the device fixation, an important aspect of device safety, as a dislocated device could obstruct smaller vessel segments downstream in the PA, requires accurate assessment of the diameters of the left and right PA as well as their respective curvatures. Furthermore, taper of the vessels’ segments, and the presence of side branches at the intended implantation site are of interest as well. These aspects are also important to model the forces of the device fixation onto the vessel wall. PAPS are usually manufactured from non-biological materials such as different polymers or metals. Furthermore, they form an obstacle for the blood flow in the PA, which results in a disturbed flow with increased turbulence, flow separation, and regions of flow recirculation. These disturbed flow features are known to promote thrombus formation especially in regions with low wall shear stresses (WSS)^17,18^.

Thus, all geometric parameters of interest for the device migration as well as the bifurcation angle between left and right PA and the enlargement of the cross-sectional area from MPA to LPA with RPA were of interest. Finally, the lengths of main left and right, as well as the main PA segments are of interest for the device implantation procedure. Table 1 summarizes motivations for the selected geometric parameters of interest in this study. Note, that the hemodynamic parameters, such as WSS or hemodynamic features, such as recirculation mentioned in the Table 1 does not represent a full list of hemodynamic parameters, which are associated with disturbed hemodynamics known to be associated with thrombus formation. A set of different hemodynamic parameters such as time-averaged WSS, oscillating shear index (OSI) or relative residence time (RRT) as well as vortical structures are parameters and features mostly associated with a prediction of the thrombosis risk by means of computational flow analysis^18–20^.

### Automated measurement of geometric size parameters

The measurements of all geometric parameters were performed automatically using a centreline-based analysis of the open-ended surfaces reconstructed from the CT image data (see Fig. 2). To generate centrelines of the segmented pulmonary arteries, we used the python library vmtk^22^. Its algorithm approximates a given surface by fitting spheres at each point inside the surface. This way, the sample points along the centreline can be determined with their corresponding radius, which is describing the averaged distance from the sample point to the vascular wall. The amount of sample points on the centreline is resampled to a consistent number for all centrelines. Also, the indices of the bifurcation points are consistently the same, allowing automated analysis.

All edges (straight short lines connecting neighbouring vertices defining the centreline) up until the first bifurcation were considered to belong to the MPA. The LPA and RPA were defined depending on the orientation within the scanner. Starting from the first edge of the LPA and RPA, at each bifurcation, the edge with the smallest angle to the previous edge was considered to belong to the LPA/RPA respectively. All other edges were defined as side branches. For each edge, the length as well as edge-averaged diameters were obtained. The measurements were accumulated to calculate the overall length and length-weighted diameters of the MPA, LPA and RPA, respectively.

### Automated measurement of geometric shape parameters

**Figure 3:**
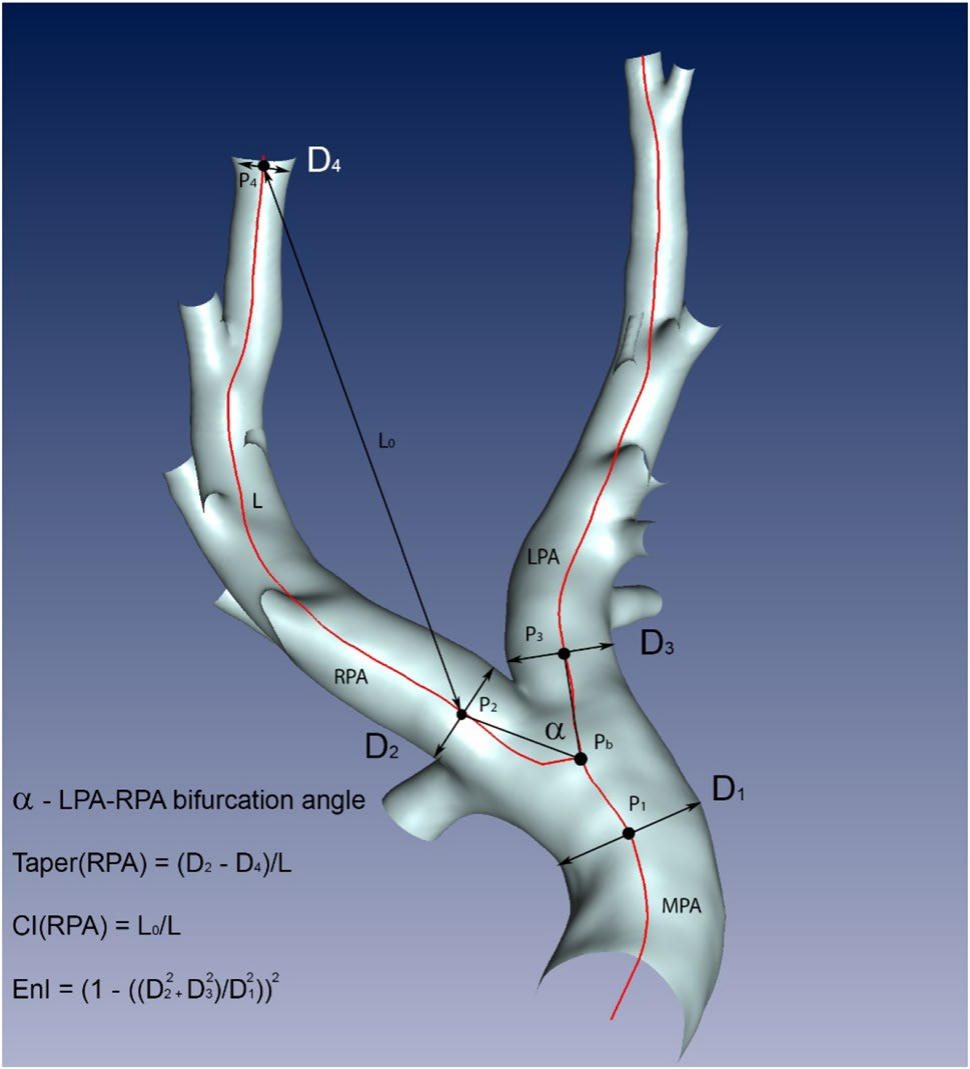
Measurements obtained from the centreline-based analysis: the centreline (red) is subdivided into several vertices, connected by edges. For each vertex the vessel diameter is available. The vertices P_2_ and P_4_ are used for the measurement of the length L, the curvature index (CI) in the RPA using the lengths L and L_0_, and the RPA taper using the diameters D_2_ and D_4_. L_0_ is the direct distance (black line with arrows) between vertices P_2_ and P_4_, whereas L is the length of the curved centreline (red line) connecting the same vertices. The vertex P_b_ marks the LPA-RPA bifurcation node. The marked vertices P_1_, P_2_ and P_3_ are located at the distance of 10 mm from the bifurcation vertex. Diameters measured at these vertices are used to measure the enlargement index EnI. The bifurcation angle α between LPA and RPA is the angle between the vectors (black lines) connecting the P_b_ with P_2_ and P_3_.

Next, parameters describing the shape complexity in a 3D space, i.e., curvature, enlargement index, taper, and the bifurcation angle, are calculated. The parameters are also described in the Table 1 and visualized in the Fig. 3. For calculation of these parameters several points of interest were specified at the centreline: the bifurcation point Pb between MPA, LPA, RPA; the end points of the LPA and RPA, and one point each on the LPA and RPA, located 10 mm downstream the bifurcation point, called P2 and P3 respectively. This downstream shift of 10 mm was chosen, as Pb was located within the vessel, while the start of the LPA and RPA should be defined using the apex of the bifurcation, i.e., when the cross sections of the LPA and RPA feature no intersections.

The curvature index was calculated from the distance of start (P2 for the RPA and P3 for the LPA) and end node divided by the overall length of the curved vessel segment. The taper was calculated based on diameter differences at the start and end node divided by the respective segment length. The enlargement index was calculated based on the diameters of the LPA and RPA at the points P2 and P3 and the diameter of the MPA at the bifurcation point. Lastly, the bifurcation angle between LPA and RPA was determined, by calculating the angle between the vectors connecting the bifurcation point and P2 and P3 at the LPA and RPA, respectively. All individual 3D reconstructions, the centrelines as well as the geometric parameters are made available as supplemental material.

### Statistical analysis

Statistical analysis was performed using IBM SPSS Statistics 28 (IBM, USA). Mean and standard deviation were reported for normally distributed parameters. Normality of distribution was assessed using a Shapiro–Wilk test. For non-normally distributed parameters, median and interquartile [IQR] range were used to report parameter distributions. A two-tailed Student’s t-Test was used to test for significant differences within normally distributed parameter differences, whereas a Mann–Whitney-U-Test and paired signed-rank Wilcoxon-Test were used for testing non-normally distributed parameter differences. To assess the agreement between both operators, Bland–Altman-analysis for the geometric parameters of interest is carried out. In addition, the intra-class correlation coefficient ICC(1,1) is calculated according to the convention by McGraw and Wong^23^. All tests used a standard significance level of 0.05.

## Results

### Operator-bias analysis

**Figure 4:**
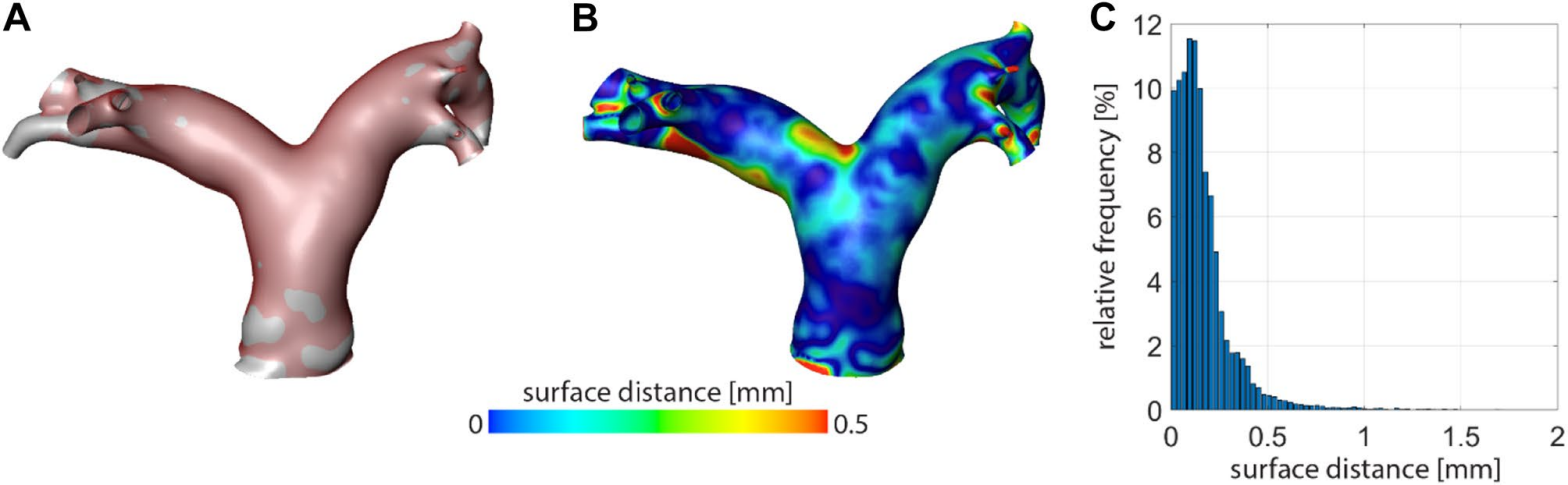
Illustration of the operator bias analysis for one exemplary use case. (**A**) Overlay of both individual surface reconstructions, using a solid grey and a transparent red surface. (**B**) Spatial resolved map of the surface distances between both individual reconstructions. (**C**) Histogram of the surface distances between both individual reconstructions.

The inter-operator analysis revealed an average surface distance between the reconstructions performed by both independent operators of 0.39 ± 0.22 mm, which equals the lower thresholds of the voxel resolution of the data sets used in this study. The accuracy of the surface reconstruction procedure is illustrated in Fig. 4 for an exemplary case.

**Figure 5:**
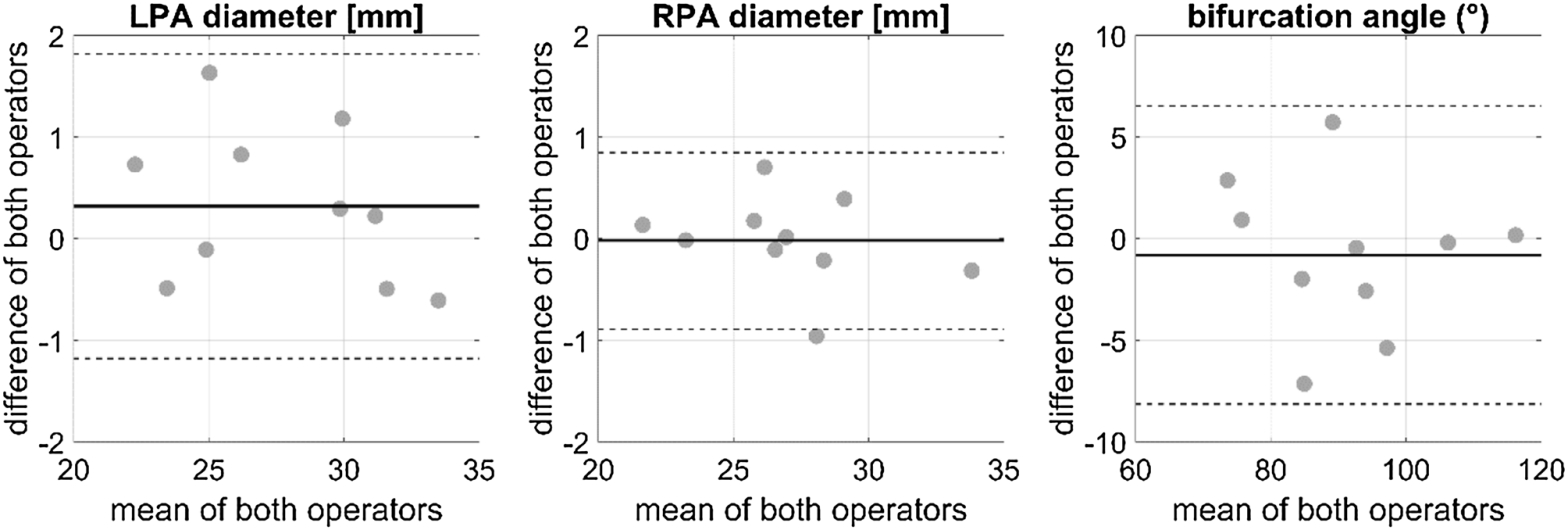
Bland–Altman-analysis for the left (LPA) and right pulmonary artery (RPA) diameter, as well as the bifurcation angle. The solid line indicates the mean of the differences between both operators, whereas the dashed lines indicate the mean ± 1.96 times the standard deviation of the difference between both operators.

LPA and RPA diameters as well as the bifurcation angle showed strong agreement between the two independent operators as indicated by Bland–Altman-analysis (see Fig. 5), as well as ICC values of 0.979, 0.992, and 0.962, respectively.

### Number of branching vessels

**Figure 6:**
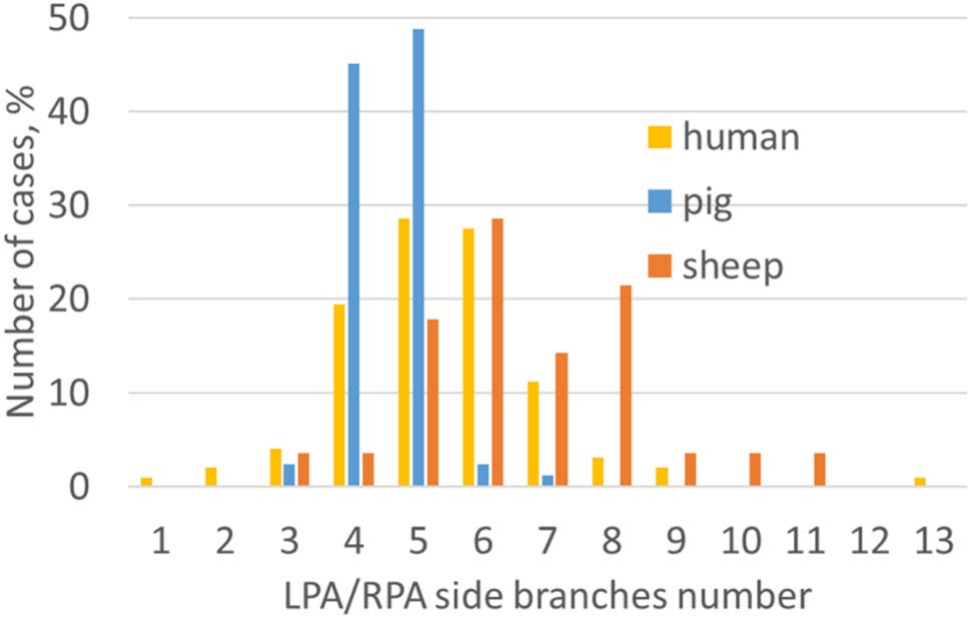
Distribution plot for the number of side branches of the LPA and the RPA vessels for all three species.

Figure 6 shows the relative frequency for the absolute number of side branches of the left or right PA found in porcine, ovine, and human data sets, respectively. Overall, the porcine PA anatomy is associated with a lower variability in the number of side branches compared with ovine and human PA. For both ovine and porcine PA, the number of the RPA side branches was significantly higher than in the LPA (paired Student’s t-Test, T = − 2.446, p = 0.029; (Wilcoxon-Test, Z = − 5.925, p < 0.001, respectively). In contrast, in human PA no significant difference was found between number of side branches in LPA and RPA (Wilcoxon-Test, Z = − 0.712, p = 0.476). Furthermore, human PA had significantly more side branches than porcine PA (Mann–Whitney-U-Test, Z = − 4.230, p < 0.001), whereas ovine PA had significantly more side branches compared to human PA (Mann–Whitney-U-Test, Z = − 5.614, p < 0.001).

### Vessel lengths and diameters

Descriptive statistic for geometric parameters of the three investigated species are provided in Table 2. No significant differences were found between human and both animal MPA lengths (Student’s t-Test, T = − 1.584, p = 0.118 for sheep and T = − 1.108, p = 0.271 for pigs). In all species the LPA was significantly shorter than the RPA (sheep: Student’s t-Test, T = 6.516, p < 0.001; pig: Wilcoxon-Test, Z = − 5.475, p < 0.001; humans: Wilcoxon-Test, Z = − 4.034, p < 0.001). The porcine RPA was significantly shorter than the human RPA (Student’s t-Test, T = 4.792, p < 0.001), whereas the human RPA was significantly shorter than the ovine RPA (Student’s t-Test, T = − 2.898, p = 0.005). The porcine LPA also was significantly shorter than the human RPA (Mann–Whitney-U-Test, Z = − 6.672, p < 0.001), whereas no significant difference was found between ovine and human LPA lengths (Mann–Whitney-U-Test, Z = − 1.504, p = 0.132). Also, no differences in lengths of LPA and RPA between male and female patients was observed (Student’s t-Test, T = 1.004 and –0.702, p = 0.321 and 0.487).

**Table 2:** Comparison of geometric parameters of porcine, ovine, and human PA. Normally distributed data are represented as mean ± SD. Otherwise, median and IQR are shown.

Parameter	Porcine	Ovine	Human
Side branches, n	5 [4, 5]	6 [5.25–8]	5 [4–6]
MPA length, mm	49.3 ± 9.83	51.5 ± 10.46	47.1 ± 8.77
RPA length, mm	100.9 ± 12.75	134.1 ± 22.76	116.9 ± 18.68
LPA length, mm	83.3 [78.1–89.1]	112.2 ± 13.95	102.0 [96.05–115.14]
MPA diameter, mm	23.4 ± 3.30	22.8 ± 6.09	27.6 ± 3.37
RPA diameter, mm	15.2 ± 2.18	12.4 ± 2.00	18.8 ± 2.45
LPA diameter, mm	14.7 [13.8–16.0]	11.9 ± 1.97	18.1 ± 1.78
LPA-RPA angle, °	71 ± 6.1	69 ± 16.2	92 ± 11.4
RPA taper, –	0.113 ± 0.0221	0.113 [0.103–0.121]	0.169 ± 0.0334
LPA taper, –	0.150 ± 0.0367	0.138 [0.124–0.160]	0.195 ± 0.0402
RPA curvature index, –	0.93 ± 0.020	0.86 ± 0.040	0.88 ± 0.053
LPA curvature index, –	0.96 ± 0.014	0.96 ± 0.017	0.77 ± 0.062
Enlargement index, –	0.07 [0.04–0.14]	0.029 [0.01–0.17]	0.53 ± 0.314

MPA diameters of both animal (sheep and pig) models were significantly smaller than those measured in humans (Student’s t-Test, T = 3.885 and 6.042, p < 0.001 for both tests). Ovine and porcine LPA and RPA diameters were significantly smaller than those measured in humans (LPA: Mann–Whitney-U-Test, Z = − 5.621 and − 6.915, p < 0.001 for both tests; RPA: Student’s t-Test, T = 4.120 and 7.293, p < 0.001 for both tests). The human LPA diameter was significantly smaller than the RPA diameter (paired Student’s t-Test, T = − 2.774, p = 0.008). Also, the porcine and ovine LPA diameters were significantly smaller than those measured in the RPA (Wilcoxon-Test, Z = − 2.780, p = 0.005 and paired Student’s t-Test, T = − 3.598, p = 0.003). No significant differences in the diameters of either vessel segment were found between male and female patients (Student’s t-Test, LPA: T = 0.985, p = 0.365, RPA: T = − 1.507, p = 0.140).

### Bifurcation angle, taper, curvature, and enlargement index

Descriptive statistic for geometric parameters of the three investigated species are provided in Table 2. The angle of the RPA-LPA bifurcation was smaller in sheep and pigs compared to that of humans (Student’s t-Test, T = 5.810 and 10.702, p < 0.001 for both tests), but no significant differences (Student’s t-Test, T = 0.446, p = 0.662) were found between the two animal species Furthermore, no significant difference was found between bifurcation angles of men and women (Mann–Whitney-U-Test, Z = − 0.965, p = 0.334).

The taper of the human RPA was significantly smaller than that of the LPA (paired Student’s t-Test, T = 7.594, p < 0.001). This was also valid for porcine (paired Student’s t-Test, T = 8.078, p < 0.001) and ovine (Wilcoxon-Test, Z = − 3.296, p = 0.001) RPA. For both LPA and RPA, taper of sheep (Mann–Whitney-U-Test, Z = − 4.398 and − 5.009, p < 0.001 for both tests) and pigs (Student’s t-Test, T = 5.391 and 9.046, p < 0.001 for both tests) was significantly smaller compared to humans.

The curvature index (CI) of the porcine and ovine LPA were significantly larger than that of the RPA (paired Student’s t-Test, T = 8.688 and 14.105, p < 0.001 for both tests), whereas the CI of the human LPA was significantly smaller than that of the RPA (paired Student’s t-Test, T = − 11.240, p < 0.001). CI of the porcine RPA as well as LPA were significantly larger than that measured in humans (Student’s t-Test, T = − 5.858 and − 20.701, p < 0.001 for both tests). CI of the ovine LPA was also significantly larger than that of measured in humans (Student’s t-Test, T = − 18.609, p < 0.001), whereas no significant difference was found between sheep and humans CI in the RPA (Student’s t-Test, T = 1.422, p = 0.160). No significant differences were found between CI measured in male and female patients either for the LPA or RPA (Student’s t-Test, T = − 0.873 and 1.349, p = 0.388 and 0.185).

The enlargement index of the human LPA-RPA bifurcation was significantly larger compared with both sheep and pigs (Mann–Whitney-U-Test, Z = − 7.304 and − 4.216, p < 0.001 for both tests), whereas no difference was found between the two animal species (Mann–Whitney-U-Test, p = 0.231). No significant difference was found between male and female patients (Student’s t-Test, T = 0.838, p = 0.406).

The similarity of animal models regarding human PA with respect to the above-mentioned measurements is summarized in Table 3. Significant differences between the animal and human anatomy are indicated by either a minus or zero, whereas a plus is used to indicate good agreement (absence of significant differences).

**Table 3:** Morphometric similarity between animal models and human PA coded by significantly different (−) or no significant differences ( +).

Parameter	Porcine versus human	Ovine versus human
Side branches	−	−
MPA/RPA/LPA length	+/−/−	+/−/+
MPA/RPA/LPA diameter	−/−/−	−/−/−
LPA diameter < RPA diameter	+	+
Angle	−	−
RPA/LPA taper	−/−	−/−
RPA taper < LPA taper	+	+
RPA/LPA CI	−/−	+/−
LPA CI < RPA CI	−	−
EnI	−	−
Similarity score	3	5

## Discussion

The comparative analysis of geometric parameters between two animal species and the human PA revealed significant differences for most of the investigated size (vessel lengths and diameters) as well as shape (taper, curvature, and enlargement indices) parameters. Overall, similarity between ovine and human PA anatomy was higher than that between pigs and humans, even though the weight of the human cohort was significantly higher than that of the ovine cohort, while the weights of the porcine and human cohorts were similar. However, both retrospective animal data sets originated from studies focussing on mimicking adult human physiology.

The observed differences in size parameters indicate that findings from both large animal models must be considered carefully. Due to the similar sizes of their overall cardiovascular anatomy, mimicking the entire implantation procedure within these animal models might be possible. However, already relevant differences in the anatomy of the implantation site and therefore in aspects of the sensor fixation might be expected.

**Figure 7:**
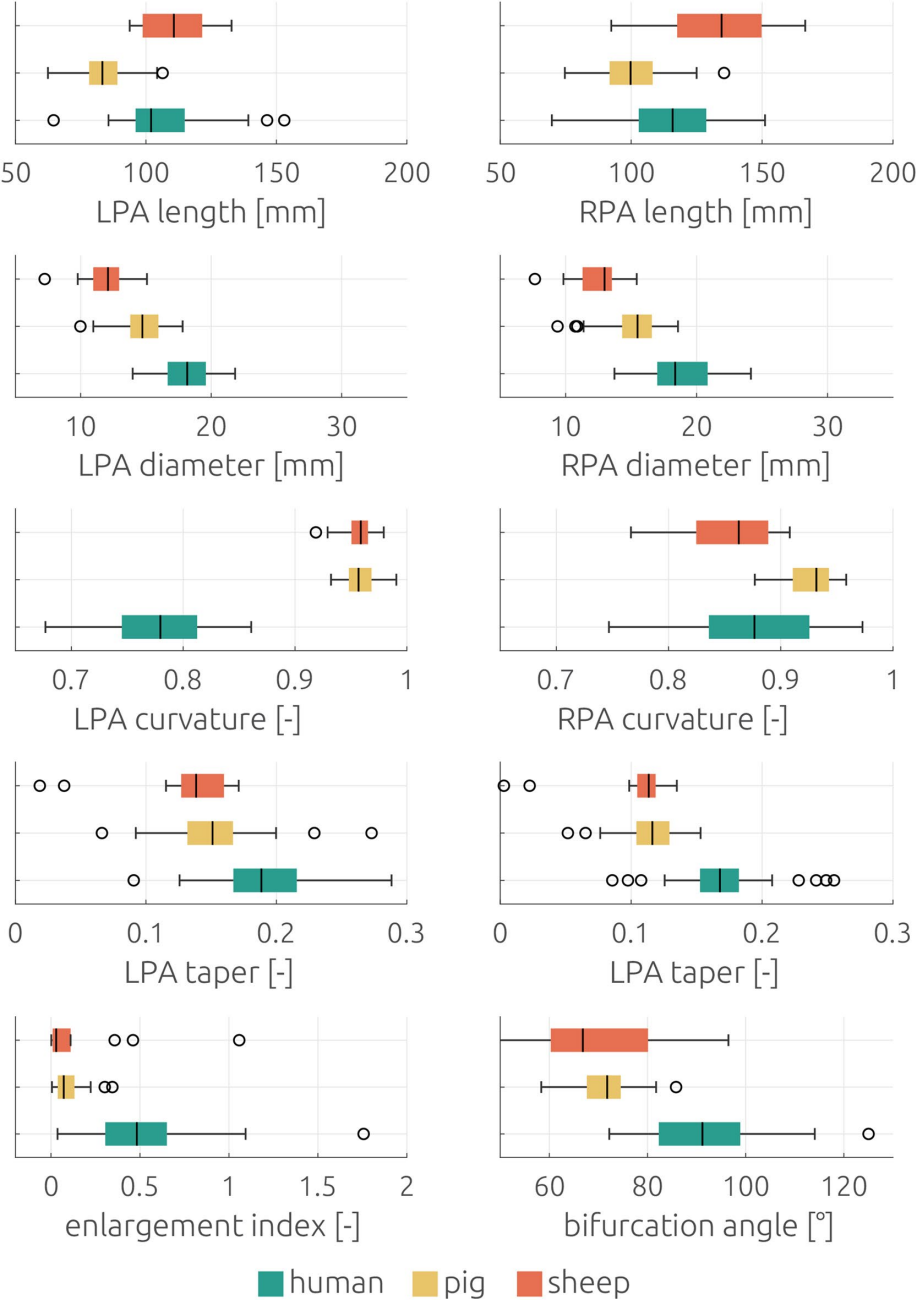
Boxplots of geometric size parameters (lengths and diameters) for all three major vessels of the pulmonary artery of the three investigated species.

For the size parameters, the parameter distributions between the different species featured a moderate overlap (see Fig. 7), indicating that a careful pre-selection of the animals might allow to identify animals with suitable vessel lengths and diameters. However, assessing the complex anatomy of the PA using imaging techniques a priori to the animal experiments is not feasible. Thus, this pre-selection must be performed on demographic aspects, such as selecting other breeds of the respective species, animals’ weight, or age. In theory, the smaller diameters observed in both animal species could be compensated by choosing older animals^11^. However, as the pulmonary artery of sheep was already longer than that of humans, this difference would increase. If controlling the anatomy a priori is not feasible and omitting the animal experiments is not viable either, the anatomy of the animals should be investigated using imaging techniques. This is necessary to at least understand the differences in the cohort investigated within the animal trial.

For several shape parameters, less overlap between the parameter distributions was observed, meaning that even with careful selection, identification of pigs or sheep with LPA curvature or enlargement indices comparable to those in humans seems impossible. Note that the set of geometric parameters investigated in this study was selected based on considerations motivated by the impact of these parameters on the proper function of the sensor to be implanted into the left or right PA. Since the shape of both animal PA features smaller curvatures, smaller bifurcation angles, smaller enlargement indices, and nearly circular cross-sections, they might also be associated with less disturbed flow compared to the human anatomy and function. Flow disturbances and recirculation regions are usually associates with hemodynamic parameters that are commonly attributed with elevated risks of thrombus formations, such as low wall shear stress values^17,18^ or higher oscillating shear indices^24^. Thus, we hypothesize that both animal models are limited in their ability to predict the risk of thrombus formation after the device implantation due to hemodynamic properties. Also, stronger heterogeneity observed with respect to the number if side branches might add to this effect, as each bifurcation causes flow disturbance^25^.

Of course, differences in thrombogenicity will not only be attributable to the differences in the hemodynamics observed in the respective species. Also, relevant differences in blood properties and biochemical processes involved in thrombogenicity are reported for ovine and porcine animal models. For example, porcine erythrocytes are smaller and stiffer than those of humans^26^ and rheologic properties differ markedly in some large animal models^27^. Furthermore, also the biochemical processes relevant for thrombus formation, such as generation of the enzymes thrombin and plasmin, feature marked differences in some animal models, such as the pig^28^. If in addition to these relevant differences, also the hemodynamics at the implant differ, translation of findings made in the animal models to human application might be encumbered further.

However, understanding the hemodynamic differences between both animal species and humans might also provide more insight into the degree to which differences in thrombogenicity can be attributed to the hemodynamics, or the animal-specific blood properties. Thus, translation of thrombogenicity experiments performed in animal models to clinical application might be strengthened. To finally judge the similarity of both animal models regarding human PA further in-vivo, in-vitro or in-silico studies are recommended. The aim of these studies should be the analysis of differences between hemodynamic parameters usually associated with risk of thrombogenicity and whether those differences can be attributed to the differences in geometric parameters presented in this study.

## Limitations

The number of investigated subjects per each species is relatively small especially for the sheep. Furthermore, reconstruction of the PA anatomy from CT image data was performed manually, which might affect the accuracy of measured geometric parameters. However, the inter-operator variability revealed small average surface distances between the independent reconstructions, which were less or equal to the average voxel size, which was about 0.6 mm. Since the spatial resolution of imaging data is the main factor affecting the accuracy of anatomy reconstruction, we consider the operator-bias to be negligible.

For automated evaluation of several parameters, including the length of the LPA and RPA, the bifurcation angle and the enlargement index, LPA and RPA centrelines where specified beginning from a point located 10 mm downstream of the bifurcation point Pb (Fig. 3). The median diameter of the MPA at Pb over all species was approximately 20 mm. Therefore, the choice of 10 mm equals roughly the MPA radius. Nonetheless, the individual starting points P2 and P3 might not perfectly located at the apex of the bifurcation. We decided for this choice in favour for a robust and automated analysis along the centreline without manual specification of LPA/RPA starting points.

Furthermore, the study was motivated, and the geometric parameters of interest were chosen based on a specific implantable cardiovascular device, which is currently only implanted seldomly. However, the findings of this study might also be helpful for future studies of other medical devices such as pulmonary heart valve prostheses^14^. Additionally, all individual reconstructions of the pulmonary artery geometries as well as the centrelines and individual parameters are made available together, allowing to calculate and assess other parameters of interest, if required for subsequent investigations.

This study investigates differences in similarities in geometric parameters based on exploration of retrospective data. We did not perform any correction for multiple comparison problems such as Bonferroni correction. Such a correction would have reduced the risk for type-I errors, identifying differences between species that are just observations by chance, while simultaneously increasing the risk for type-II errors, ruling out relevant differences. Which error type is to be preferred is not trivial to answer and, as this will directly address the question of the validity of large animal models, might be affected by subjective aspects. Furthermore, the retrospective study design did not allow for proper a-priori power analysis. However, as we provide all individual measurements, additional statistical evaluations can easily be performed if required for specific questions of interest.

Therefore, we decided against using any correction method but to provide an analysis of the expected effects assuming we would have used Bonferroni correction. For the comparison of the geometric parameters between the three species, most calculated p-values were below 0.001, which would still be considered significant in a study design with 50 hypothesis tests. Only the comparison of the length in the ovine and human RPA resulted in a p-value of 0.005, which would not have been considered significant after Bonferroni correction. In this case, the similarity between human and ovine PA would have been rated even stronger, strengthening the statement that the ovine animal model is better suitable than the porcine one, while simultaneously still showing relevant differences in other parameters.

In contrast to the human patients, CT scans of both animal species were performed during general anaesthesia. Some of the pharmaceuticals used, such as propofol, are known to cause vasodilation, which could affect diameter measurements in the animals. As diameters in both animals were smaller than in humans, this effect could potentially even be larger without anaesthesia resulting in less good agreement.

Finally, our morphometric analysis was limited to the end-diastolic phase. Assessing the same geometric parameters also during peak-systole, calculating the differences between end-diastolic and peak-systolic phases for all three species, as well as additional parameters resulting from this analysis, such as the distensibility, are of great interest. Due to the retrospective nature of this study, this analysis was not possible as most of the CT data sets did not include transient information of the anatomy.

## Conclusion

This comparative analysis of morphometric parameters in the ovine, porcine, and human PA provides relevant information for the choice of an appropriate animal model for preclinical studies for assessing safety and efficacy of cardiovascular devices to be implanted into the pulmonary artery. Based on our study, geometrical similarity between ovine and human pulmonary arteries are stronger than between porcine and human anatomy. Thus, the sheep might be better suitable to test device implantation procedures as well as hemodynamically moderated processes such as thrombogenicity. However, future studies between animal models and humans should also be extended by functional analysis of differences between hemodynamic parameters to also allow quantification of the hemodynamic similarity or the lack thereof. Finally, this study’s findings as well as the data provided together with it might hopefully help to reduce the number of animal experiments in future preclinical studies as well as reduce costs and accelerate the certification procedure for novel medical devices targeting the pulmonary artery.

## Data Availability

All individual surface geometries of the human, porcine, ovine pulmonary arteries as well as the morphometric parameters are made available together with this study: https://doi.org/10.6084/m9.figshare.21919428. Further data sets used and/or analyzed during the current study are included in this published article and available from the corresponding author on reasonable request. The repository is not yet published thus, the DOI is not yet active, but the data can be accessed via the private link provided below for reviewers.

## References

[1] Khoo, S.Y., Justifiability and animal research in health: Can democratisation help resolve difficulties? Animals (Basel). 2018. **8**(2). 10.3390/ani8020028 PMC5836036 29443894

[2] Russell WMS, Burch RL (1959). The principles of humane experimental technique.

[3] Plessis J, Hascoet S, Baruteau A, Godart F, Le Gloan L, Warin Fresse K, Tahhan N, Riou JY, Guyomarch B, Petit J, Guerin P (2018). Edwards SAPIEN transcatheter pulmonary valve implantation: Results from a French registry. JACC Cardiovasc. Interv..

[4] Shahanavaz S (2020). Transcatheter pulmonary valve replacement with the Sapien prosthesis. J. Am. Coll. Cardiol..

[5] Mullens W, Sharif F, Dupont M, Rothman AMK, Wijns W (2020). Digital health care solution for proactive heart failure management with the Cordella Heart Failure System: Results of the SIRONA first-in-human study. Eur. J. Heart Fail.

[6] Assmus B, Angermann CE, Alkhlout B, Asselbergs FW, Schnupp S, Brugts JJ, Nordbeck P, Zhou Q, Brett ME, Ginn G, Adamson PB, Bohm M, Rosenkranz S (2022). Effects of remote haemodynamic-guided heart failure management in patients with different subtypes of pulmonary hypertension: Insights from the MEMS-HF study. Eur. J. Heart Fail.

[7] Dong M, Yang W, Tamaresis JS, Chan FP, Zucker EJ, Kumar S, Rabinovitch M, Marsden AL, Feinstein JA (2020). Image-based scaling laws for somatic growth and pulmonary artery morphometry from infancy to adulthood. Am. J. Physiol. Heart Circ. Physiol..

[8] Burrowes KS, Hunter PJ, Tawhai MH (2005). Anatomically based finite element models of the human pulmonary arterial and venous trees including supernumerary vessels. J. Appl. Physiol..

[9] Burrowes KS, Hoffman EA, Tawhai MH (2009). Species-specific pulmonary arterial asymmetry determines species differences in regional pulmonary perfusion. Ann. Biomed. Eng..

[10] Lee YC, Clark AR, Fuld MK, Haynes S, Divekar AA, Hoffman EA, Tawhai MH (2013). MDCT-based quantification of porcine pulmonary arterial morphometry and self-similarity of arterial branching geometry. J. Appl. Physiol..

[11] Lipiski M, Eberhard M, Fleischmann T, Halvachizadeh S, Kolb B, Maisano F, Sauer M, Falk V, Emmert MY, Alkadhi H, Cesarovic N (2020). Computed Tomography-based evaluation of porcine cardiac dimensions to assist in pre-study planning and optimized model selection for pre-clinical research. Sci. Rep..

[12] Shavelle D, Jermyn R (2016). The CardioMEMS heart failure sensor: A procedural guide for implanting physicians. J. Invasive Cardiol..

[13] Guichard, J.L., F. Sharif, O. Forouzan, J. Martina, and L. Klein, A procedural guide for implanting the cordella pulmonary artery pressure sensor. J. Invasive Cardiol. (2022). 10.25270/jic/22.00209 36562797

[14] Spriestersbach H, Prudlo A, Bartosch M, Sanders B, Radtke T, Baaijens FP, Hoerstrup SP, Berger F, Schmitt B (2017). First percutaneous implantation of a completely tissue-engineered self-expanding pulmonary heart valve prosthesis using a newly developed delivery system: A feasibility study in sheep. Cardiovasc. Interv. Ther..

[15] Franke B, Bruning J, Yevtushenko P, Dreger H, Brand A, Juri B, Unbehaun A, Kempfert J, Sundermann S, Lembcke A, Solowjowa N, Kelle S, Falk V, Kuehne T, Goubergrits L, Schafstedde M (2021). Computed tomography-based assessment of transvalvular pressure gradient in aortic stenosis. Front. Cardiovasc. Med..

[16] Kuprat A, Khamayseh A, George D, Larkey L (2001). Volume conserving smoothing for piecewise linear curves, surfaces, and triple lines. J. Comput. Phys..

[17] Turitto VT, Hall CL (1998). Mechanical factors affecting hemostasis and thrombosis. Thromb. Res..

[18] Hyun S, Kleinstreuer C, Archie JP (2000). Hemodynamics analyses of arterial expansions with implications to thrombosis and restenosis. Med. Eng. Phys..

[19] Zambrano BA, Gharahi H, Lim CY, Lee W, Baek S (2022). Association of vortical structures and hemodynamic parameters for regional thrombus accumulation in abdominal aortic aneurysms. Int. J. Numer. Method Biomed. Eng..

[20] Jiang X, Cao H, Zhang Z, Zheng T, Li X, Wu P (2022). A hemodynamic analysis of the thrombosis within occluded coronary arterial fistulas with terminal aneurysms using a blood stasis model. Front. Physiol..

[21] Batchelor CK, Batchelor G (1967). An introduction to fluid dynamics.

[22] Izzo R, Steinman D, Manini S, Antiga L (2018). The vascular modeling toolkit: A python library for the analysis of tubular structures in medical images. J. Open Source Softw..

[23] McGraw KO, Wong SP (1996). Forming inferences about some intraclass correlation coefficients. Psychological Methods.

[24] Park, M.H., Y. Qiu, H. Cao, D. Yuan, D. Li, Y. Jiang, L. Peng, T. Zheng. Influence of hemodialysis catheter insertion on hemodynamics in the central veins. J. Biomech. Eng. 142(9) (2020) 10.1115/1.4046500 32110795

[25] El-Masry OA, Feuerstein IA, Round GF (1978). Experimental evaluation of streamline patterns and separated flows in a series of branching vessels with implications for atherosclerosis and thrombosis. Circ. Res..

[26] Amin TM, Sirs JA (1985). The blood rheology of man and various animal species. Q. J. Exp. Physiol..

[27] Windberger U, Bartholovitsch A, Plasenzotti R, Korak KJ, Heinze G (2003). Whole blood viscosity, plasma viscosity and erythrocyte aggregation in nine mammalian species: Reference values and comparison of data. Exp. Physiol..

[28] Tarandovskiy ID, Shin HKH, Baek JH, Karnaukhova E, Buehler PW (2020). Interspecies comparison of simultaneous thrombin and plasmin generation. Sci. Rep..

